# Recent advances in the understanding and management of mucormycosis

**DOI:** 10.12688/f1000research.15081.1

**Published:** 2018-09-07

**Authors:** Benoit Pilmis, Alexandre Alanio, Olivier Lortholary, Fanny Lanternier

**Affiliations:** 1 Paris Descartes University, Sorbonne Paris Cité, Infectious Diseases Unit, Necker-Enfants Malades University Hospital, AP-HP, Imagine Institute, Paris, France; 2 Antimicrobial Stewardship Team, Microbiology Unit, Groupe Hospitalier Paris Saint Joseph, Paris, France; 3 Université Paris Diderot, Sorbonne Paris Cité, Laboratoire de Parasitologie-Mycologie, Hôpital Saint-Louis, Groupe Hospitalier Lariboisière, Saint-Louis, Fernand Widal, AP-HP, Paris, France; 4 Molecular Mycology Unit, Institut Pasteur, CNRS UMR2000, Paris, France; 5 Centre National de Référence Mycoses invasives et Antifongiques, Institut Pasteur, Paris, France

**Keywords:** Mucormycosis, Reverse Halo sign, PCR, Isavuconazole, Liposomal amphotericin B

## Abstract

Mucormycoses were difficult-to-manage infections owing to limited diagnostic tools and therapeutic options. We review here advances in pathology understanding, diagnostic tools including computed tomography, and serum polymerase chain reaction and therapeutic options.

## Introduction

Mucormycoses are life-threatening fungal infections mostly occurring in hematology, solid organ transplant, or diabetic patients, it may also affect immunocompetent patients following a trauma or burn ^1^. Nosocomial or community outbreaks have been described ^2^. Mucormycosis is characterized by host tissue infarction and necrosis resulting from vasculature invasion by hyphae starting with a specific interaction with endothelial cells. Most common clinical presentations are rhino-orbito-cerebral and pulmonary. Multicenter and single-center studies have reported an increasing incidence probably due to an increase in the at-risk population and improved diagnostic tools ^3, 4^. In a French study, mucormycosis incidence increased by 7.3% per year, especially in patients with neutropenia ^5^.

These infections are difficult to manage for several reasons ^6^. Firstly, diagnosis is difficult because of clinico-radiological similarities with invasive aspergillosis and historical lack of diagnostic tools. However, new tools in serum and tissue as well as the recognition of highly suggestive radiological signs recently modified diagnostic possibilities. Secondly, treatment is an emergency and combines surgery, which is frequently required owing to the angioinvasive and necrotic character of infection ^7^, and antifungal treatment. Primary *in vitro* resistance to several antifungal drugs limits therapeutic options ^8^. However, recent data enlarge the antifungal armamentarium with the US Food and Drug Administration’s and European Medicines Agency’s approval of the new triazole isavuconazole. However, comparative clinical data are lacking, and the respective places of polyenes and different azoles need to be discussed.

## Moving towards improved mucormycosis understanding

**Figure 1.**
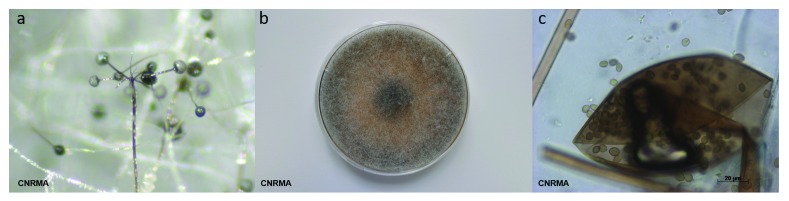
Typical features of *Rhizopus arrhizus*. ( **a**) Sporangiophore branching and rhizoids (stereomicroscope); ( **b**) grey-brownish colony on malt 2% medium; ( **c**) melanized sporangium and sporangiospores.

Human mucormycoses are caused by a wide range of pathogenic species. Mucormycosis location is linked to the *mucorales* species; *Rhizopus arrhizus* ( Figure 1) is present in 85% of rhino-cerebral forms, compared with only 17% of non-rhino-cerebral forms in the French RetroZygo study ^9^. This finding could be explained by virulence differences between *Mucorales* species. In experimental studies, ketoacidosis has been found to predispose mice to *Rhizopus* spp. but not *Lichtheimia spp.* infection ^10, 11^. In parallel, corticosteroid treatment enhanced the susceptibility of mice to lung infections caused by *Lichtheimia corymbifera* or *Lichtheimia ramosa* ^10, 11^.

Mucormycosis’ clinical presentation is also related to underlying conditions. Rhino-cerebral mucormycosis is the most common form in patients with diabetes mellitus ^1^, while pulmonary mucormycosis occurs most often in patients with hematological malignancies ^12^. Radiological findings in patients with pulmonary mucormycosis are also related to immunological status ^13^. Although unusual, lately there have been diagnoses made in the gastrointestinal system. The stomach is more frequently involved, and then the colon. Symptoms are abdominal pain and gastro-intestinal bleeding ^14^. Diagnosis is suspected on endoscopic findings with necrotic lesions that can lead to perforation and peritonitis ^15^.

*Mucorales* can gain entry to a susceptible host through inhalation, ingestion of contaminated food, or abraded skin. These routes result in rhino-orbito-cerebral, pulmonary, gastrointestinal, or cutaneous/wound infections. One of the characteristic features of mucormycosis is its angioinvasive property, resulting in vascular thromboses and ultimately tissue necrosis. Ketoacidosis and deferoxamine are known to predispose to mucormycosis, revealing the importance of hyperglycemia, iron, and acidifying ketone bodies in *mucorales* virulence. Angioinvasion was reported to be related to the interaction between a spore-coating protein family (CotH) on *Rhizopus* spp. surface and endothelium glucose regulator protein 78 (GRP78) expressed at the surface of endothelial cells. This interaction triggers host cell injury and subsequent fungus hematogenous dissemination ^16^. Elevated levels of serum glucose, iron, and ketone bodies increase fungal growth and induce the expression of GRP78 and CotH, resulting in increased ability of *Rhizopus* to invade host tissues and explaining the susceptibility of diabetic and deferoxamine-treated patients to mucormycosis. However, it should be noted that the majority of studies on virulence and the association between ketoacidosis and the occurrence of mucormycosis have been conducted with *Rhizopus* species ^17^. Decreased numbers and impaired function of monocytes and neutrophils are important mucormycosis risk factors, since they are known to inhibit *Mucorales* spore germination. This includes patients with hematological disorders, AIDS, or liver cirrhosis, those who have undergone solid organ transplant, and those being treated with high-dose steroids ^18, 19^. Finally, victims of natural disaster are also at risk ^20^ owing to wounds contaminated with water, soil, or debris ^21^, such as after the 2004 Indian Ocean tsunami ^22^ or after the 2011 Missouri tornado ^23^.

## Computed tomography

**Figure 2.**
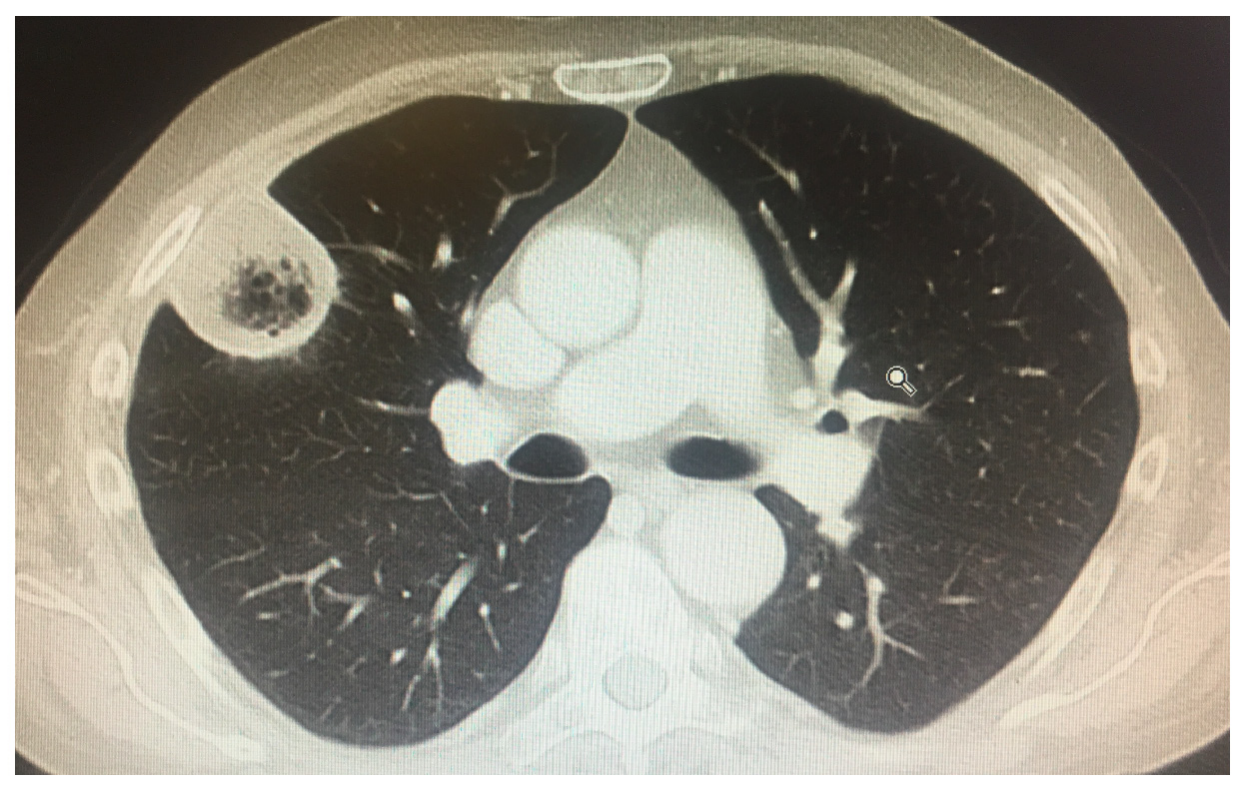
Inversed halo sign. Inversed halo sign.

The most common radiological pattern of lung mucormycosis on initial computed tomography (CT) scan is a halo sign and then nodule or mass ^13, 24^. However, when studied very early and on serial follow-up, sequential morphologic changes could be observed as (i) reversed halo sign ( Figure 2) followed by (ii) consolidation or nodule or mass with halo sign and, finally, (iii) central necrosis and air-crescent sign. For pulmonary mucormycosis, a recent study showed that there was a significant increase in the prevalence of reversed halo sign in neutropenic (79%) and non-neutropenic (31%) patients ( *P* <0.05) ^25^.

## Major steps in mucormycosis: towards earlier diagnosis ( Figure 3)

Mucormycosis diagnosis is challenging, as it is associated with high mortality, especially in hematological patients. Early distinction from invasive aspergillosis is of utmost importance, as antifungal treatment may differ, whereas underlying conditions and clinical presentation are often similar. Until recently, mucormycosis diagnostic tools were based on limited basic microbiology and frequently led to diagnosis delay.

**Figure 3.**
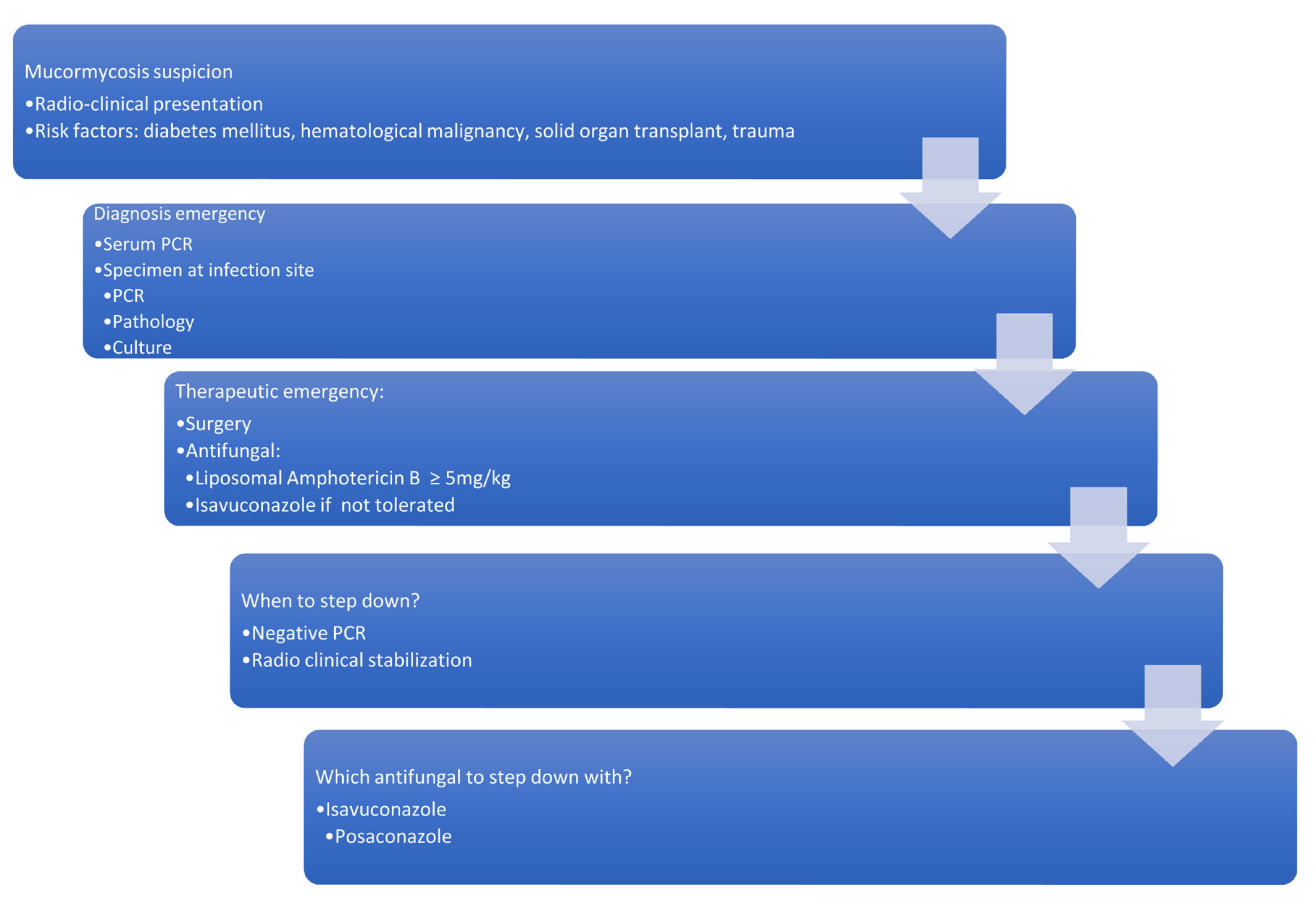
Mucormycosis: from suspicion to treatment. PCR, polymerase chain reaction.

Unlike invasive aspergillosis, the detection of circulating antigen such as galactomannan and β-D-1,3-glucan provides no help for mucormycosis diagnosis. Therefore, samples from the infection site are highly required to diagnose mucormycosis based on the microscopic detection of typical hyphae or on a positive culture ^26^. Recently, the development of molecular biology tools has allowed the non-invasive diagnosis of mucormycosis. Million *et al.* designed a quantitative multiplex polymerase chain reaction (qPCR)-based 18S rRNA targeting *Mucor/Rhizopus*, *Lichtheimia*, and *Rhizomucor*. This PCR assay was evaluated with the aim to detect *Mucorales* DNA early in the course of the infection in the blood (serum) ^27^. The authors were able to detect *Mucorales* DNA in serum samples from 90% of patients up to three days before mucormycosis diagnosis ^27^. Negative serum PCR was also associated with better outcome as compared to patients with persistently positive PCR. Furthermore, a study among severely ill burn patients found that circulating *Mucorales* DNA was detected 11 (4.5–15) days before standard diagnosis for invasive wound mucormycosis ^28^. Other studies have evaluated the use of real-time PCR targeting *Mucorales* on tissue or respiratory samples in patients with hematological malignancy suffering from proven and probable mucormycosis ^29– 32^.

Consequently, it is currently necessary in patients with hematological malignancies to include the value of reverse halo sign on CT combined with serum qPCR targeting *Mucorales* in the early diagnosis of pulmonary mucormycosis ^33^.

## Several antifungals now available for mucormycosis treatment

### Mucormycoses: an indication of emergency surgery

Current guidelines recommend antifungal treatment, surgical debridement, and correction of risk factors ^34, 35^. Surgical debridement has to be extensive, involving all necrotic areas for rhino-oculo-cerebral infection, and repeated surgical procedures are recommended to achieve local control and improve outcome ^36^. For pulmonary mucormycosis, the indication and timing of surgical management outside emergency care (hemoptysis) is still unclear ^37^. In a European series of 230 patients, surgical treatment reduced mortality by 79% ^38^, leading to discuss surgery when feasible for any localization, however mandatory for rhino-cerebro-oculo-cerebral and post-traumatic mucormycosis ^36, 39^.

### Antifungal treatment

Amphotericin B (Amb) and its lipid formulations and posaconazole were the only antifungal drugs available with *in vitro* activity against *mucorales* ^40, 41^. The antifungal armamentarium recently enlarged with the development of isavuconazole.

The first-line recommended antifungal agent is liposomal Amb (L-Amb) or Amb lipid complex (ABLC) ^35^. Studies in mice proposed that the efficacy of L-Amb and ABLC was dependent on the dose given and that 10 mg/kg yielded the best outcomes. A prospective French phase II multicenter study (AmBizygo trial) evaluated the efficacy and tolerance of high-dose (10 mg/kg/day) L-Amb in association with surgery when recommended for the treatment of 34 mucormycosis cases. A favorable response was seen in 45% of patients at week 12. However, serum creatinine doubled in 40% of patients, but in 63% of cases, once treatment had ended, creatinine levels normalized after three months ^42^. According to this study, ECMM/ESCMID and ECIL-6 guidelines recommend the use of L-Amb with a daily dosage of at least 5 mg/kg/day for mucormycosis ^34, 35^, and dosages at 10 mg/kg/day are strongly supported by ECMM/ESCMID for cerebral infections ^35^. Moreover, because of better diffusion, L-Amb should be favored in central nervous system infections.

The duration of the first-line antifungal treatment is still a matter of debate and should be determined on an individual basis and adjusted based on the underlying condition. Some authors proposed a lipid Amb treatment for at least three weeks, and, when there is clinical and radiological improvement, a consolidation by posaconazole can be started ^43^. However, it could possibly be guided by negative PCR and therefore shortened for some patients.

Isavuconazonium sulfate is a water-soluble pro-drug, which is quickly hydrolyzed to the triazole isavuconazole after oral or intravenous administration. Isavuconazole has high oral bioavailability, linear pharmacokinetics, and a broad antifungal spectrum. The *in vitro* activity of isavuconazole minimum inhibitory concentration (MIC) ranges were 0.125 to 4 mg/L across *L. corymbifera*, *L. ramosa*, *Rhizomucor pusillus*, *Rhizomucor microspores*, and *R. arrhizus* but somewhat higher against *Mucor circinelloides* (1 to 16 mg/L). The MICs were in general one- to three-fold higher than those for posaconazole ^44^. In the recently published Vital study, 21 patients were treated with isavuconazole as first-line treatment; 42-day response rate was only 14% and week 12 response was 10% (compared to 45% in the AmBizygo study) with 43% deaths ^45^. The results of this study found a mortality rate at day 42 comparable to that observed in the AmBizygo study ^42^. In the VITAL study, isavuconazole was well tolerated and toxic effects were an uncommon cause of discontinuation. The place of isavuconazole has not yet been specified in the most recent guidelines ^34^. Finally, a cost-effectiveness study demonstrated the positive economic impact of the use of isavuconazole compared to Amb in the treatment of mucormycosis ^46^.

Posaconazole has been shown to have *in vitro* and *in vivo* activity against *mucorales*, but there are no data for the use of first-line posaconazole therapy. Posaconazole, therefore, finds its place in the therapeutic armamentarium for prophylaxis or consolidation after induction treatment with L-Amb. No study on the efficacy of posaconazole intravenous or tablet formulations in mucormycosis treatment were conducted. Finally, mucormycosis cases have been reported in patients undergoing posaconazole prophylaxis despite satisfactory serum concentrations ^47^.

However, it seems important to note that there are no current validated MIC breakpoints for any of the available antifungals and thus the determination of susceptibility categories is not possible for the agents of mucormycosis.

### Immunostimulating drugs

A case report has recently reported the benefit of a treatment with the checkpoint inhibitor nivolumab and interferon-Υ for an immunocompetent patient with extensive abdominal mucormycosis unresponsive to conventional therapy ^48^.

## Conclusion

Mucormycosis is a life-threatening fungal infection characterized by host tissue infarction and necrosis that occurs mostly in immunocompromised patients and is associated with an increasing incidence and mortality despite the availability of therapeutic tools. Determining whether the patient has invasive aspergillosis or mucormycosis could be challenging at the bedside. In this context, new tools of molecular biology have been developed to obtain earlier diagnosis and start optimal medico-surgical treatment. Comparative studies are needed to better optimize induction and consolidation treatment.
